# A Multi-Compartment Hybrid Computational Model Predicts Key Roles for Dendritic Cells in Tuberculosis Infection

**DOI:** 10.3390/computation4040039

**Published:** 2016-10-21

**Authors:** Simeone Marino, Denise E. Kirschner

**Affiliations:** Department of Microbiology and Immunology, University of Michigan Medical School, Ann Arbor, MI 48109, USA

**Keywords:** agent-based model, multi-compartmental model, tuberculosis, dendritic cells, uncertainty and sensitivity analysis

## Abstract

Tuberculosis (TB) is a world-wide health problem with approximately 2 billion people infected with *Mycobacterium tuberculosis* (Mtb, the causative bacterium of TB). The pathologic hallmark of Mtb infection in humans and Non-Human Primates (NHPs) is the formation of spherical structures, primarily in lungs, called granulomas. Infection occurs after inhalation of bacteria into lungs, where resident antigen-presenting cells (APCs), take up bacteria and initiate the immune response to Mtb infection. APCs traffic from the site of infection (lung) to lung-draining lymph nodes (LNs) where they prime T cells to recognize *Mtb*. These T cells, circulating back through blood, migrate back to lungs to perform their immune effector functions. We have previously developed a hybrid agent-based model (ABM, labeled *GranSim*) describing in silico immune cell, bacterial (Mtb) and molecular behaviors during tuberculosis infection and recently linked that model to operate across three physiological compartments: lung (infection site where granulomas form), lung draining lymph node (LN, site of generation of adaptive immunity) and blood (a measurable compartment). Granuloma formation and function is captured by a spatio-temporal model (i.e., ABM), while LN and blood compartments represent temporal dynamics of the whole body in response to infection and are captured with ordinary differential equations (ODEs). In order to have a more mechanistic representation of APC trafficking from the lung to the lymph node, and to better capture antigen presentation in a draining LN, this current study incorporates the role of dendritic cells (DCs) in a computational fashion into *GranSim*.

**Results:**

The model was calibrated using experimental data from the lungs and blood of NHPs. The addition of DCs allowed us to investigate in greater detail mechanisms of recruitment, trafficking and antigen presentation and their role in tuberculosis infection.

**Conclusion:**

The main conclusion of this study is that early events after Mtb infection are critical to establishing a timely and effective response. Manipulating CD8+ and CD4+ T cell proliferation rates, as well as DC migration early on during infection can determine the difference between bacterial clearance vs. uncontrolled bacterial growth and dissemination.

## 1. Introduction

Tuberculosis (TB) remains one of the main causes of death world-wide and the leading cause due to an infectious disease [1]. For such an ancient disease, it is surprising that so little is still known about what provides a protective response against infection with *Mycobacterium tuberculosis* (Mtb), the causative agent. When infection occurs with Mtb, two main outcomes are observed. One is active disease where the host is unable to contain infection, which if left untreated results in death of the host (about 5%–10% of those infected). Active disease can occur directly after infection (primary TB), after reactivation (see below) or in the case of re-exposure (which is probably the most common pathway leading to disease in highly endemic countries). The difference between re-exposure and re-activation likely plays a role in the immune response observed. The second outcome is latent infection. This occurs when the host controls infection, which remains clinically latent even though bacteria are still harbored (about 90% of infected) [2]. Latent infection can become reactivated if the host is compromised in some way leading to active disease. There is still no efficacious vaccine against Mtb, although ~30 vaccines are in various stages of testing and clinical trials (http://www.aeras.org/). Long regimens of antibiotics (6–9 months) with multiple drugs are needed to control infection. Antibiotics also represent a double-edged sword, since they lead to Mtb resistance (which is rapidly increasing), especially due to long time regimens that are naturally associated with non-compliance. New treatment and prevention strategies are desperately needed to make a major impact on TB morbidity and mortality. However, the host-pathogen interactions occurring during Mtb infection are complex and span across multiple biological scales, ranging from bacterial and cellular to organ to an entire host, making research on TB challenging.

When Mtb bacteria are inhaled into lungs, they are taken up by two types of lung resident immune cells that are known generally as antigen-presenting cells (APCs): these are macrophages (MΦs) and dendritic cells (DCs). Mtb is preferentially an intracellular pathogen, however their growth rate is extremely slow compared to most bacteria (days rather than minutes). APCs are typically unable to kill Mtb unless they are in a highly activated state, and thus bacteria grow and burst out of these cells, killing their host cell; and are taken up by new APCs. This process continues, leading to the development of the hallmark of Mtb infection: a granuloma. Granulomas are a collection of host immune cells (e.g., macrophages, DCs and T cells) together with bacteria and infected cells, with a centralized necrotic region. It is presumed that the organization is an attempt to contain or eliminate the infection, but Mtb have evolved mechanisms that permit survival within granulomas. Within a single host, several granulomas form in response to the initial infection dose, and these granulomas are heterogeneous with variable trajectories, complicating the study of this infection [3–5]. For example, in some hosts none of the granulomas are successful at controlling bacterial replication, and those that fail lead to a pattern of dissemination and new granuloma formation, resulting in lung destruction and active TB. In other hosts, granulomas can all be successful and the host can develop latent infection. Thus infection dynamics play out at the scale of granuloma. T cells play a central role in protection against TB [6–11], as best exemplified by the dramatic susceptibility of HIV+ humans to TB, even in the early stages of HIV infection [12–14]. Other immune cells are increasingly shown to play key roles in the immune dynamics of Mtb infection and T cells are interdependent on their dynamics.

**Figure 1:**
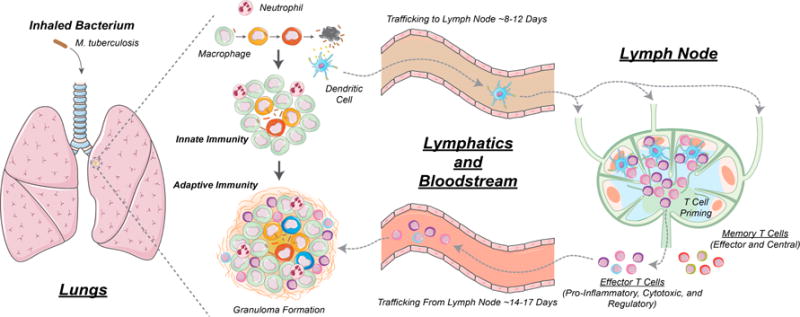
Overview of the immune response to *Mycobacterium tuberculosis* (Mtb) infection. Infection begins in lungs and antigen-presenting cells (APCs) such as dendritic cells (DCs) take up Mtb and then traffic from lungs to lung draining lymph nodes (LNs) where they prime T cells via the process of antigen presentation. This occurs when pieces of Mtb (called antigens) are presented on the surface of dendritic cells (DCs) to T cells to activate T cells. These T cells migrate back to the lungs via blood, and participate in granuloma formation and function, including functions such as activation of macrophages to kill their intracellular Mtb [9,15]. Some T cell subsets that have been primed by DCs (cytotoxic CD8+ T cells) can kill infected macrophages directly [11,16,17].

What has received far less attention are the cells of the early immune response in Mtb infection, e.g., DCs, and it is likely that these cells bridge to long-term immunity in important and key ways. Figure 1 shows how dynamics occurring in lungs, lymph nodes and blood are dynamically linked and each participates in the host-pathogen interactions describing Mtb infection. Most experimental studies focus on a single biological (length and/or time) scale of interest, e.g., examination of immune cells in blood or a particular signaling pathway. To truly understand the complex in vivo immune response to Mtb, it is important to integrate information from experiments performed at multiple scales and over multiple physiological compartments (lung, blood, lymphatics, and lymph nodes). To address this complex disease we thus need a comprehensive and integrative tool to generate testable hypotheses about what characterizes an effective immune response to Mtb infection. We use a mathematical and computational modeling approach to identify key features of the host immune system that can serve as targets for control of infection. We focus specifically on the role of dendritic cells as they serve as the link between physiological compartments of lungs and lymph nodes (LNs) that generate activated immune cells that can traffic to lung granulomas to aid in infection control.

Mathematical/computational models are powerful tools for deciphering the outcomes of multiple simultaneous, nonlinear processes. In particular, agent-based models (ABMs) link molecular and cellular behavior—and therapeutic interventions aimed at molecules and cells—with tissue scale outcomes, such as a growing or stable granulomas or containment of infection. Excellent reviews on ABMs can be found in [15–18]. Because we are interested in individual cellular behavior, ABMs are the appropriate modeling type here.

While we have modeled the host-Mtb response using non-linear ordinary differential equation (ODE) systems [19–23], we and others have built ABMs that capture both the spatial and temporal dynamics of granuloma formation in the lung [20,22,24–28]. Our modeling framework, *GranSim*, focuses on immune dynamics in the context of bacterial dynamics is a hybrid agent-based model (for full details see [29]). We have used *GranSim* to explore drug treatment during Mtb infection [30–32] and performed virtual clinical trials to predict optimal treatment strategies [30–35]. Here we explore a version of *GranSim* that is multi-compartment, where the hybrid ABM is connected to two non-linear systems of ODEs tracking compartmental models capturing dynamics of blood and lung draining LNs. This multi-compartment model was recently used to explore the existence of biomarkers for TB [36]. However in that model, we only had a mathematical phenomenological proxy for APCs moving from the lung to the LN and similarly a probability function capturing recruitment of T cells from the LN back to the granuloma. Here, we replace the phenomenological expressions for these processes by explicitly including DCs in the lung model *GranSim*, and tracking their trafficking from lung to LN where they orchestrate priming of T cells. We use our sensitivity and uncertainty analyses techniques to analyze the 3-compartmental hybrid system and identify which mechanisms are driving different granuloma outcomes in the lung [37]. In addition, we derive a way to scale our single granuloma lung model to a whole host scale so that we are not only able to calibrate our model with data, but also so we are able to compare our results with data derived from non-human primates (NHP) as we have done previously [36]. Our predictions can be used to predict how certain treatments could improve infection outcomes.

## 2. Materials and Methods

### 2.1. GranSim: Computational Model of Granuloma Formation and Function in the Lung

The pathologic hallmark of Mycobacterium tuberculosis (Mtb) infection in humans and NHPs is the formation of spherical structures, primarily in the lungs, called granulomas. Infection occurs after inhalation of Mtb into the lungs. Resident antigen presenting cells (APCs) such as MΦs and DCs, take up Mtb and initiate granuloma formation. DCs traffic to lung-draining LNs where T lymphocytes are primed. These T lymphocytes migrate to the lung and participate in granuloma formation and function (see Figure 1). We have developed a hybrid agent-based model (ABM, labeled *GranSim*) describing in silico cellular (i.e., macrophages and T cells), bacterial and molecular behaviors during Mtb infection in three physiological compartments: lung (site of infection), draining lymph node (LN, site of generation of adaptive immunity) and blood (a measurable compartment) [36].

Our computational model captures single granuloma formation and function in the lung [24,25,38,39], while LN and blood compartments [19,40] represent dynamics of the whole body in response to infection, i.e., we assume they are well-mixed compartments.

*GranSim* captures cellular recruitment to lungs, chemotaxis of cells, changes of cell states (activation, infection, etc.), cytokine and chemokine secretion, as well as effector T cell functions [24,25,38,39,41]. Probabilistic interactions between immune cells and bacterial populations are described by sets of rules between immune cells and Mtb in the lung that are updated based on new biological data [39,42,43]. We also capture multi-scale events, such as tumor necrosis factor (TNF) or interleukin-10 (IL-10) receptor/ligand binding and trafficking and intracellular signaling events with ordinary differential equations (ODEs) that are solved within each agent [38,39,41–43]. Diffusion of relevant chemokines and cytokines is performed by solving the relevant partial differential equations (PDEs) [44]. Each simulation follows events over several hundred days, building over time to track thousands of individual cells (agents). Based on our recent work [32], we represent the section of lung tissue where granuloma typically develops with a larger spatial grid (4 × 4 mm) to better capture physiological granuloma sizes (with mean and standard deviation of 2 and 0.5 mm, respectively, on a collection of ~500 granulomas, see [32] for details). This new, larger grid size comprises a collection of 200 × 200 micro-compartments sized to a macrophage diameter of ~20 mm. All of the rules and an executable file for *GranSim* can be found at [29].

### 2.2. Multi-Compartment Gransim: Tracking Cell Dynamics in the Lymph Node and Blood

Our unique multi-scale and multi-physiological compartmental, hybrid computational model generates in silico data on dynamics of infection in both blood and lymph node, capturing formation of independent granulomas in lungs and at the same time T cell profiles in blood. In a recent study [36], we easily captured LN and blood dynamics using a compartmentalized system of non-linear 31 ordinary differential equations (ODEs), where we tracked CD4+ and CD8+ T cells with different memory classes (i.e., Naïve, Effector, Central and Effector Memory), both Mtb-specific and non Mtb-specific. Mtb-specific T cells represent a generic class of antigen-specific T cells, assuming that all Mtb-specific antigens are equally immune-responsive. This system of ODEs can be found in the Supplementary File 1.

### 2.3. Adding DCs to GranSim

In order to have a better representation of APC trafficking from the lung to the lymphatics, herein we added a new class of cells to *GranSim*, namely dendritic cells (DCs). DCs are considered professional APCs, since their main task is to sample tissues and blood for foreign cells/non-self particles/antigen and, when needed, to traffic to specific organ draining lymph nodes to initiate a specific immune response by presenting their findings to T cells. The initial number of resident DCs in lungs is based on a fraction (*percentOfMacInitNumber*) of resident macrophages in the lung, and consequently on the grid. These numbers are calibrated from experimental staining of healthy lung tissues in our previous studies [20,22]. DC recruitment to the lungs is executed with a random probability of *percentOfMacInitNumber*. Once on the grid, we assume that a DC moves and secretes cytokines and chemokines similarly to [45,46].

The first major difference between macrophages and DCs in our model is that macrophages can take up Mtb and kill their intracellular load, while it has been shown that DCs do not kill their intracellular burden [47–49]. Macrophages can do this task more efficiently when stimulated by cytokines such as INF-γ. Another major difference between macrophage and DC dynamics in our model is that once a DC interacts directly (or indirectly) with Mtb antigens, it is labeled as “stimulated”. Mtb antigen stimulation can occur in the following ways: (i) DCs uptake Mtb; (ii) Infected Macs are in the DC in the Moore Neighborhood (i.e., defined as the grid spaces on a two-dimensional square lattice that are composed of a central grid space and the eight grid spaces that surround it) [50]; (iii) Extracellular Mtb are in the Moore Neighborhood of a DC and (iv) Dead Mtb are in the Moore Neighborhood (i.e., the surrounding 8 grid compartments of a given grid space in the ABM) of the DC.

Once DCs are stimulated, a parameter determines the time of DC exit from the lung and allows it to migrate into the lymphatics (*exitInterval*), which is the conduit to the draining LN. In contrast, macrophages never exit the grid, they can only die (via age or by being killed a number of different ways (Apoptosis, induced cell death by cytoxic T cells, etc.). The lymphatics are represented in the model as a virtual compartment to mimic spatial delays during DC trafficking from the site of infection to the LN. After exiting the lung, DCs are placed in a queue, where another parameter (*exitToLN*) tracks the physiological delay observed for DCs to reach the draining lymph node. This delay is observed on average to be about a few days to a week in most infections [51]. Once in the LN, DCs perform antigen presentation leading to T cell priming and activation as described in [19,36].

### 2.4. Scaling to Host Feature

When a host is infected with Mtb, not one, but a large number of granulomas form in the lungs over time. The median number of granulomas at 4 weeks was 46 ± 21 (range 13–97, *n* = 14 monkeys) [52]. This number is due to the bacterial dose that the host receives, and also the ability of bacteria to disseminate in the lung. *GranSim* currently captures granuloma formation and function of a single granuloma during TB infection in the lung. We now explicitly introduce a scaling factor (i.e., *scalingMDC*) to capture TB infection in the whole lung. In other words, we multiply the number of DCs migrating to the LN from our single granuloma model by *scalingMDC* to represent multiple granulomas draining DCs into the LN. This larger number of DCs is then passed to the ODE system representing the LN-Blood compartments, where the DC Equation (S1) is pulsed accordingly (see more details below). We calibrate this scaled *GranSim* (that is coupled to the LN and blood ODE model) to experimental data derived in the lung and the blood for each non-human primate (NHP). The blood data is available longitudinally, while the lung data is taken from many different NHP at the time of death (necropsy) over different time, that we collate into a time series (see section below on NHP data for a full explanation and also [19]).

We assume that the majority of the granulomas found in the lung at necropsy (i.e., the parameter *scalingMDC*) have developed at the time of initial infection. The scaling to host step is performed by multiplying the number of stimulated DCs in our in silico granuloma by *scalingMDC*. The resulting quantity pulses Equation (S1) of the system of ODEs, namely the equation capturing DC dynamics (see Supplementary File 1 for all the details on the equations cited in the manuscript).

**Figure 2:**
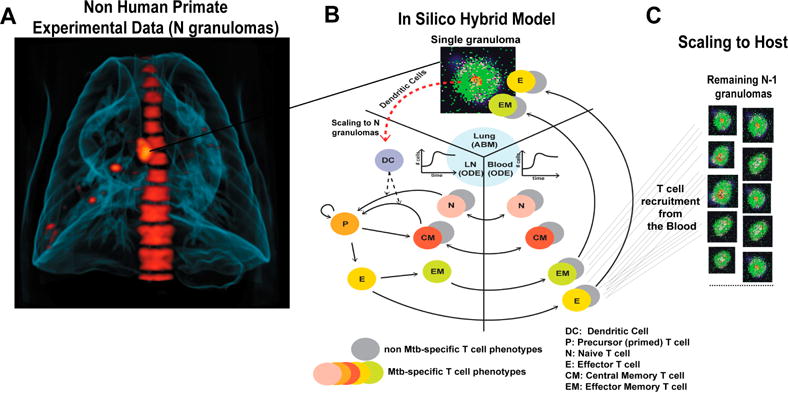
Scaling to host methodology. Our in silico model captures single granuloma formation in the lung. Panel (**A**) shows a PET-CT scan of the lung of an infected Non-Human Primate (NHP). An important and likely independent driver of Mtb infection outcome is inflammation. In vivo 18F-Fluorodeoxyglucose (FDG)-PET/CT signals are used to measure the extent of inflammation both in humans [3] and in non-human primates infected with Mtb [52,53]. PET-CT scan is an advanced nuclear imaging technique which combines positron emission tomography (PET) and computed tomography (CT) into one machine. A PET/CT scan reveals information about both the structure and function of cells and tissues in the body during a single imaging session. FDG is a PET probe that incorporates into metabolically active host cells. FDG avidity is calculated by standardized uptake values (SUVs), a measure of the metabolic activity of each granuloma and is corrected for granuloma size [53]. The red (“hot”) spots represent inflammation within granulomas indicating a number of granulomas are present (image courtesy of Joanne Flynn lab). A diagram of our in silico multi-compartment hybrid model is shown in Panel (**B**). An Agent-Based Model captures formation of a single granuloma in the lung, while a system of 31 ordinary differential equations (ODEs) captures the lymph node coupled to the blood dynamics of the whole host. Panel (**C**) illustrates the scaling to host methodology implemented to capture recruitment to the other granulomas. Where N−1 granuloma remain in the lung with the Nth being the one we model with GranSim.

Figure 2 shows an example of the scaling to host procedure with *scalingMDC* = **N**. Antigen presentation (see Equations (S2) and (S11)) and T cell priming (see Equations (S3)–(S6) and (S12)–(S15)) are then performed in the lymph node compartment and many different T cell phenotypes are generated and migrate from LN into the blood. Some of these T cells traffic through blood and reach the site of infection. This is driven by chemokine gradients and many signals induced by infection and inflammation in the lung. Since we only model one granuloma in detail (i.e., *GranSim*), we recruit Effector-(E) and Effector Memory-(EM) T cells to *GranSim* first and update T cell levels in blood. Then, we perform recruitment N−1 times to mimic recruitment in the remainder of the granulomas in the lung. Recruitment in these N−1 granulomas is performed assuming similar recruitment conditions at their vascular sources. At the end of each recruitment step, the blood levels are updated reflecting the number of E- and EM-T cells that have successfully migrated to the other granulomas.

### 2.5. Uncertainty and Sensitivity Analysis

We quantify the importance of each host mechanism involved directly and indirectly in the infection dynamics using statistical techniques known as uncertainty and sensitivity analyses. In our recent published review on uncertainty and sensitivity (US) analyses techniques [54], we showed how multidimensional parameter spaces can be globally sampled in a computationally efficient manner by Latin hypercube sampling (LHS) algorithms. Correlations between model output and parameter values can then be determined using Partial Rank Correlation Coefficient (PRCC), which varies between −1 (perfect negative association/correlation between model output and parameters) and +1 (perfect positive association/correlation between model output and parameters). A PRCC value of zero or close to zero can be interpreted as having no (significant) association/correlation between model output and parameters. Statistical tests are available to assess whether a PRCC is significantly different from 0, as well as if two PRCCs are significantly different (see [37] for a complete review). In this work we specifically address time-dependent correlations that can be tracked by plotting time courses of significant PRCCs with respect to many outputs classified as contributing to inflammation, infection, adaptive immune response and blood/lymph node factors. By combining both of these analysis tools [55–58], we guide our understanding as to how and what extent variability in model mechanisms captured by parameter values can affect infection outcomes in an ordered fashion. We have successfully used this approach in our previous studies, both equation-based (i.e., ordinary, partial and delay differential equation systems), as well as agent-based model settings [24,25,59–61].

Our computational model is a hybrid model which combines a deterministic system of ordinary differential equations with a stochastic agent-based model. Thus we need to address both epistemic (or subjective, reducible, type B uncertainty, see [62]) and aleatory (or stochastic, irreducible, type A) uncertainty (see [37,62] for details). Epistemic uncertainty is driven by input/parameter variation, which is assumed to be constant throughout the in silico simulation. Aleatory uncertainty emerges anytime stochastic inputs/parameters are built into an in silico simulation. Thus, unless the random generator selects the same seed, a stochastic model will always generate different outcomes.

To address epistemic uncertainty we perform 1000 parameter sweeps (i.e., parameter samples), while aleatory uncertainty is addressed by performing 10 replications for each parameter sweep/combination. This yields 10,000 replications of the model that gives us a solid basis for analysis. We then calculate PRCCs with respect of the many outcomes under investigation on the mean of the 10 replicates to control for random effects and aleatory uncertainty.

Given the multi-compartmental nature of our system, detailed uncertainty and sensitivity (US) analyses were applied to our model to explore model dynamics both within the same compartment (intra-compartmental/intra-scale) and between different compartments or physiological scales (inter-compartmental/inter-scale). Here, we vary 50 parameters total: 8 initial conditions for T cell memory phenotypes in the blood, 36 parameters in the LN-Blood compartment and 6 parameters in the lung compartment (see Tables 1 and 2 for the parameters varied and the ranges we used).

We examined the following 16 time points (shown as days post infection) during infection progression, chosen to match the time points of the NHP blood data samples, namely 1, 10, 20, 30, 42, 50, 56, 60, 70, 84, 90, 100, 111, 140, 167 and 200 days. A list of outputs analyzed with the correspondent sensitivity coefficients is shown in Tables 3–5, as well as in Figures 5–7.

### 2.6. Experimental Data: Non Human Primate Lung and Blood Data

For the purpose of model calibration both in the lung and blood compartments, we used the dataset described in our recent work [19]. Briefly, for the blood compartment, a total of 58 cynomolgus macaques (*Macacca fasicularis*) or non-human primates (NHPs) were previously infected with a low dose of Mtb (Erdman strain, ~25–50 CFU). Blood samples were taken from 28 NHPs at the following time-points: pre Mtb infection and at days 10, 20, 30, 42, 56, 90 (or M3, 3 months), 120 (or M4), 150 (or M5) and 180 (or M6) post infection. Levels of T cells were measured, and stratified by CD4+ and CD8+ memory sub-populations based on the surface markers on the cells (expression of CD45RA and CD27, namely Naïve-N [CD45RA+ CD27+], Central Memory-CM [CD45RA− CD27+], Effector Memory-EM [CD45RA− CD27−] and Terminally Differentiated-TD or Effector-E [CD45RA+ CD27−]) For experimental data in the lung, numbers of granulomas and numbers of bacteria (referred to as colony forming units, or CFUs) per granuloma were collected at necropsy from 43 NHPs. See Supplementary Files 2 and 3.

## 3. Results

The results will be presented in two main parts. First we show how the updated model was calibrated to the experimental data to ensure the model is appropriate for study. In the second part we use uncertainty and sensitivity analysis methodologies applied to the comprehensive model to investigate and predict mechanisms that drive infection and other outcomes during the interplay between Mtb and the host.

### 3.1. Model Calibration—Lung and Blood Compartments

**Figure 3:**
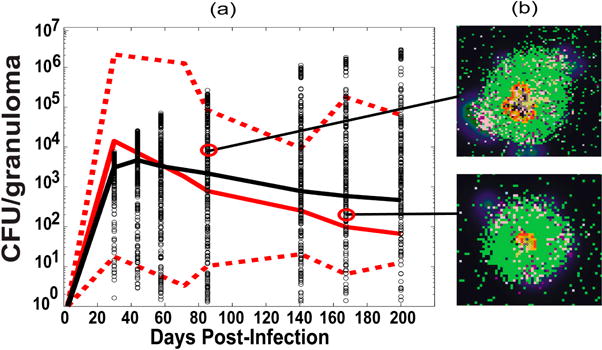
Computational model calibration: LUNG. (**a**) Time courses of CFU per granuloma. In red are shown NHP experimental data (median, max and min) for colony-forming-units (CFU)/granuloma (see details in the Supplementary File 2. They are plotted here versus our in silico datasets (black) of CFU/granuloma (lung compartment) from our computer simulations of 3000 granulomas coupled to the blood and LN dynamics). The *x*-axis shows a time span of infection up to 200 days to match the NHP blood data. The *y*-axis represents bacteria levels as CFU/granuloma. The in silico dataset of time courses of CFU/granuloma generated in the lung compartment (black circles, with the black solid line representing the median trajectory) are plotted together with experimental data on NHP CFU/granuloma (with the solid red line representing the median, and the dotted red lines representing the min and max values in the NHP data). The median trajectories for both the NHP and in silico data are calculated including the granulomas that cleared infection (Mtb < 1), while the min trajectories excluded them; (**b**) Two snapshots of in silico granuloma corresponding to the points in the time courses of panel (a)

Our granuloma models were developed, calibrated and validated using extensive data primarily from NHPs and humans, and where lacking, from mice [21,24,25,34,36,38,39,41,63,64]. We calibrated the current model to NHP experimental data in the lung (i.e., bacteria known as colony forming units, or CFU per granuloma) and blood (memory T cell levels). Tables 1 and 2 show the ranges used to generate our in silico dataset of 3000 model simulations of CFU dynamics in the lung as well as T cell dynamics in the blood. Figure 3a shows our model calibration to experimental data on the number of bacteria (given as CFU, or colony-forming-units) per granuloma from the lungs of NHPs [32,38,42]. *GranSim* also provides the ability to not only track temporal dynamics of cells and molecules but also their spatial distribution, which can be validated directly by experimental data that are also provided from NHP granulomas. This allows for comprehensive spatial and temporal investigation of mechanisms driving the heterogeneity and variability that is observed in granuloma types and their corresponding outcomes (see Figure 3b for examples of multiple in silico granuloma snapshots taken from the 3000 simulations and Figure 3 from [36] for examples of a comparison between a lung granuloma from an NHP with one generated from *GranSim*).

**Figure 4:**
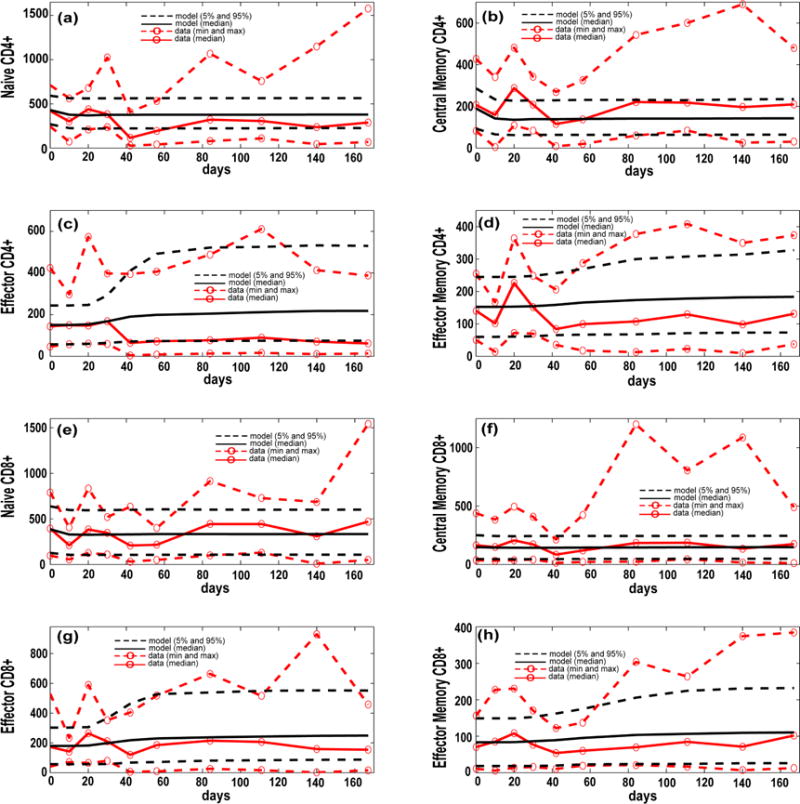
Computational model calibration (blood compartment). NHP experimental data on blood T cell phenotypes (see Supplementary File 3) are plotted here versus the in silico datasets of blood T cell phenotypes (blood compartment), from our computer simulations of 3000 granulomas coupled to the blood and LN dynamics. The *x*-axis shows a time span of infection up to 200 days to match the NHP blood data. The *y*-axis represents cells/cm^3^. (**a**–**h**) In silico dataset of 3000 time courses of 8 T cell classes generated in the blood compartment (black solid line [mean] and black dashed lines [5th and 95th percentiles]) compared to experimental data on T cell phenotypes in the blood of Mtb-infected NHPs (red dashed lines with red open circles, representing the min and max). For the minimum and maximum of the NHP data we chose the lowest and highest values at any time point across all the NHPs. In silico predictions are displayed as median (black solid line) and minimum and maximum (dashed black lines). We show Naïve CD4+ T cells (**a**) and CD8+ T cells (**e**); Central Memory CD4+ T cells (**b**) and CD8+ T cells (**f**); Effector CD4+ T cells (**c)** CD8+ T cells (**g**) and Memory CD4+ T cells (**d**) and CD8+ T cells (**h**). The in silico data have been obtained by summing the respective Mtb-specific and non Mtb-specific equations of the blood compartment of the computational model [36].

The current computational model was also calibrated with respect to blood T cell dynamics as measured in [36] (see Figure 4). Blood NHP experimental data are compared to median, 5th and 95th percentiles of our 3000 model simulations in the blood/LN compartments. Due to the limited and extremely variable NHP dataset that was available from the blood compartment, minimum and extremely variable NHP dataset that was available from the blood compartment, minimum and maximum ranges at each time point were chosen across all the subjects in order to establish the boundaries of our model simulations. Both the interpretation and accuracy of the measures of these different memory phenotypes in vitro are still being assessed (see [36] for a complete discussion of the uncertainty and variability associated with the NHP blood data and the current state-of-the-art in terms of cell profiling and Mtb-specificity). Currently the variability associated with each blood measure is not quantifiable experimentally (experiments by our collaborators are in progress in order to give us a better understanding of the experimental pure error for each blood assay). Thus, our major goal in calibrating blood dynamics was to ensure that our in silico simulations fell reasonably within the general behavior of the data (e.g., medians), rather than reproducing its large variability (e.g., min/max). Figure 4 illustrates how the model recapitulates the experimental data of host cell classes. Specifically, the predictions for the median trajectories of the Central Memory phenotypes are only affected for the maximum ranges. This can be explained by our uncertainty analysis assumptions. We assumed, a priori, uniform probability density functions for all the parameters and initial conditions that we varied (see Uncertainty Analysis section), thus we were forced to use conservative ranges for the Central Memory initial conditions in order to place the model median initial condition close to the median of the experimental data.

### 3.2. Bacterial, CD4 and CD8 Proliferation Impact Infection Burden at the Granuloma Site

After the model was adequately calibrated to the experimental data, we used it to ask questions about mechanisms playing key roles in immune protection, controlled inflammation, and in general adaptive immune response magnitude and timing during infection. Inflammation is associated with an immune response that is mounted in response to an infection. Typically, once infection is reduced and cleared, inflammation subsides. To adequately investigate mechanisms driving infection and inflammation, we perform uncertainty and sensitivity analysis (US/A) on many outcomes of the model (see Appendix B) at different times during the simulations, from the early onset (first 2 months), up to 200 days post infection. The results for the main mechanisms driving infection (e.g., total bacterial burden, or infected cells) are shown in Table 2.

US/A results support a key role for T cell priming and proliferation (in the lymph node) in mounting a protective immune response to Mtb infection. In particular, by increasing CD8+ T cell proliferation we can impact a large spectrum of host and pathogen immunological events, from total levels of infection (e.g., bacterial numbers in the lungs, total infected cells, …) and inflammation, to granuloma size and how much central caseation is present in granulomas. Table 2 highlights mechanisms/parameters that we found to be significantly associated with changes in infection correlates, such as total numbers of infected macrophages or numbers of dendritic cells, total bacteria numbers and granuloma size. Not surprisingly (as a positive control), higher bacterial numbers and numbers of infected macrophages emerge from increasing bacterial growth rates (intracellular). Increasing rates of CD4+ and CD8+ T cell proliferation, as well as rates of CD8+ T cell priming in LN have a positive impact on total bacteria in lung (lower levels). The latter three mechanisms exemplify the concept of *inter-compartmental/inter-scale* effects, where mechanisms operating in one compartment/organ (LN in our case) are affecting outcomes in a different compartment/organ (lung in our case). On the other hand, the significant effect of bacterial growth rate on outcomes clearly illustrates an *intra-compartmental/intra-scale* effect.

**Figure 5:**
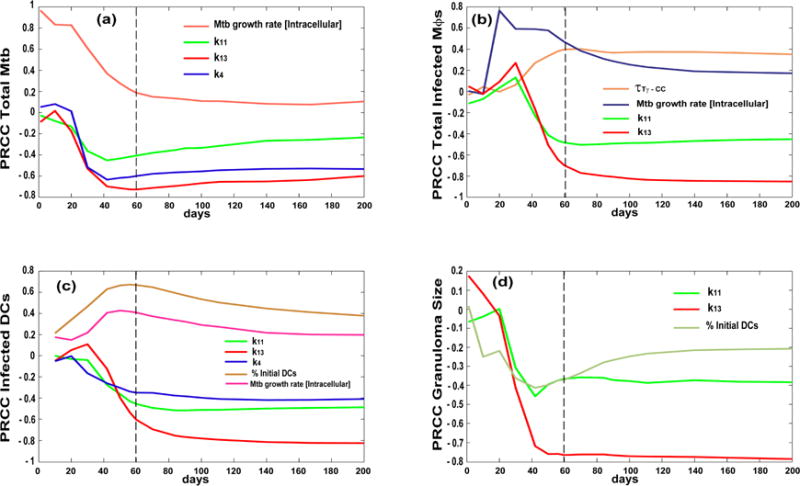
Time courses for Partial Ranked Correlation Coefficient (PRCC) of mechanisms/parameters affecting infection outcomes as they relate to Table 2. Each curve plotted is days post infection (up to 200 days) on the *x*-axis and PRCC values on the *y*-axis (that vary between −1 and 1). The PRCCs plotted are only ones that were significant (i.e., *p* < 10^−3^) and with an absolute value greater than 0.3. Outcomes shown are (**a**) total Mtb, (**b**) total infected macrophages, (**c**) total infected dendritic cells and (**d**) granuloma size. Compare with Table 2 results. Parameter definitions: k_4_ [CD4+ T cell precursor proliferation in the LN], k_13_ [CD8+T cell precursor proliferation in the LN], $\tau_{T_{\gamma }-\mathrm{CC}}$:chemokine threshold for Tγ cells recruitment to the lung, τ_Treg−TNF_: TNF threshold for Treg cells recruitment to the lung, k_11_: Naïve CD8 priming (see Appendix A for details on the parameters).

Figure 5 shows time courses of the sensitivity indexes (i.e., PRCCs) for some of the outputs in Table 2. A vertical dotted line on the plots represents the early events (i.e., first 2 months post infection). Some mechanisms/parameters have a significant PRCC only early on during infection (e.g., intracellular bacterial growth rate in Figure 5a), while some elicit their regulatory effects only late during infection (e.g., CD8+ T cell priming [k_11_] in Figure 5b). Interestingly, the CD8+ T cell precursor proliferation rate [k_13_] changes their impact on the granuloma size over time (Figure 5d).

### 3.3. Priming and Proliferation in the LN Drives Inflammation at the Site of Infection

**Figure 6:**
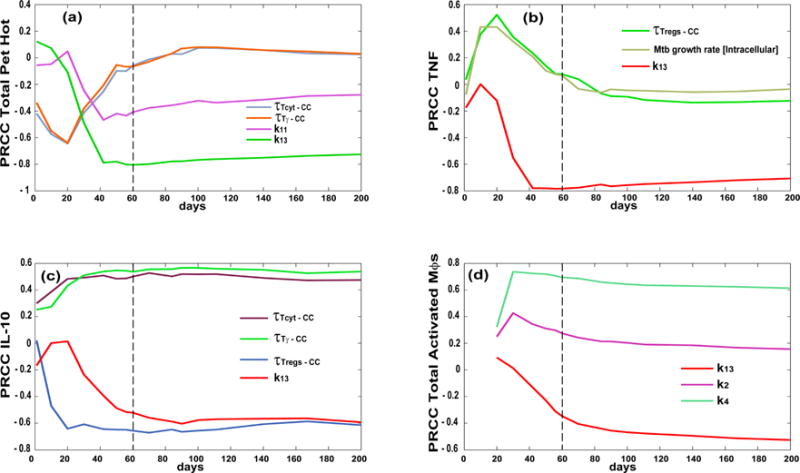
Partial Ranked Correlation Coefficient (PRCC) time courses of mechanisms/parameters affecting inflammation outcomes. Each plot has days post infection (up to 200 days) on the *x*-axis and PRCC values on the *y*-axis (between −1 and 1). The PRCCs plotted are the only ones that resulted significant (i.e., *p* < 10^−3^) and with an absolute value greater than 0.3. Outcomes shown are **a**) total Pet Hot, (**b**) TNF, (**c**) IL-10 and (**d**) total activated macrophages. Parameter definitions: $\tau_{T_{\gamma }-\mathrm{CC}}$—chemokine threshold for Tγ recruitment, T_cyt−CC_—chemokine threshold for Tcyt recruitment, τ_Treg−CC_—chemokine threshold for Treg recruitment, k_2_—Naïve CD4+ T cell priming, k_4_—CD4+ T cell precursor proliferation, k_13_—CD8+ T cell precursor proliferation, k_14_—CD8+ T cell differentiation to effector, k_11_—Naïve CD8+ T cell priming (see Appendix A for details on the parameters).

Inflammation is when many immune cells and molecules are recruited and secreted at a site of infection. This is a double-edged sword in most infections where the influx of mediators is helpful to control infection; however, too much inflammation can cause damage to the host and so must be tightly regulated. Here, we have many ways to represent inflammation in the model. Table 3 and Figure 6 showcase different outputs that we track over time that are associated with pro- and anti-inflammatory events at the site of infection of the lung. Table 3 shows sensitivities associated to total macrophage activation, total Pet Hot (a proxy for metabolically active sites as measured through PET CT scan, see Figure 2 legend herein and for details [53,65]), tissue damage (caseation/necrosis), a pro-inflammatory molecule (Tumor Necrosis Factor—TNF) and an anti-inflammatory molecule (Interleukin 10—IL-10). Typically it is thought that inflammation in tuberculosis, and most diseases, is associated with infection. However Table 3 shows only a marginal direct effect of bacterial growth rate on inflammation. This suggests the host is mediating most of the inflammation observed.

While higher CD4+ T cell proliferation rates (i.e., k_4_) in the LN compartment are naturally associated with higher levels of macrophage activation (i.e., a necessary step in macrophage activation), higher CD8+ T cell proliferation rates (i.e., k_13_) have a general anti-inflammatory role, likely due to the higher levels of killing of infected cells and lower levels of bacteria. However, the higher cytotoxicity, likely associated with higher CD8+ T cell proliferation rates, results in greater tissue damage, as shown by the strong positive correlation between k_13_ and higher levels of central caseation/necrosis within granulomas. It is interesting to note how the levels of IL-10 (a typically anti-inflammatory molecule) are strongly affected by the different effector T cell chemokine thresholds for recruitment at the site of infection as compared to TNF, which is more of a pro-inflammatory molecule. Figure 6 shows a more comprehensive picture of the impact of many of these mechanisms on inflammation dynamics during infection, emphasizing the timing aspect as well: some are important early (e.g., chemokine threshold for recruitment, Figure 6a,b) versus later in infection progression (e.g., k_13_ in Figure 6b–d).

### 3.4. T Cell Priming, Proliferation and Trafficking Determine the Timing and Magnitude of the Immune Response at the Site of Infection and in the Blood

**Figure 7:**
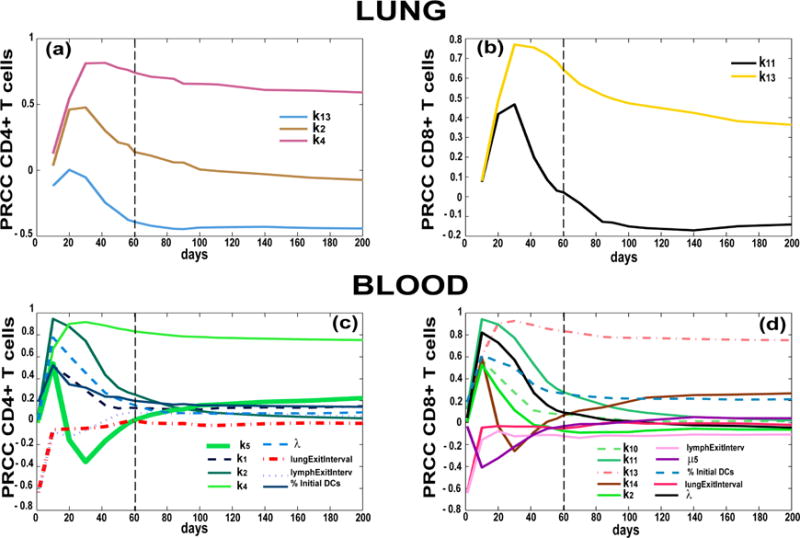
Partial Ranked Correlation Coefficient (PRCC) time courses of mechanisms/parameters affecting adaptive immune response in the lung and blood compartments. Each plot has days post infection (up to 200 days) on the *x*-axis and PRCC values on the *y*-axis (between −1 and 1). The PRCCs plotted are the only ones that resulted significant (i.e., *p* <10^−3^) and with an absolute value greater than 0.3. Outcomes shown are Mtb-specific Effector T cells in the lung ((**a**) CD4+ T cells and (**b**) CD8+ T cells) and in the blood ((**c**) CD4+ T cells and (**d)** CD8+ T cell). Parameter definitions: k_2_—Naïve CD4+ T cell priming, k_4_—CD4+ T cell precursor proliferation, k_13_—CD8+ T cell precursor proliferation, k_14_—CD8+ T cell differentiation to effector, k_11_—Naïve CD8+ T cell priming, λ: Frequency of Mtb-specific Naïve T cells in the blood/LN, _μ5_: half-life of Mature DCs in the LN (see Appendix A for details on the parameters.)

A protective immune response is one where not only is the bacteria cleared or strongly contained, but where damage to the host, induced by too much inflammation, is controlled. Using our US/A, we characterized key mechanisms driving a protective immune response at the site of infection by tracking Mtb-specific T cells as well as dendritic cell dynamics in the lung (see Table 4). CD8+ T cell proliferation (k_13_) shows up again with very strong correlations across all outcomes. It is interesting to note how T_γ_ and T_cyt_ seem to be complementary: high CD8+ T cell proliferation rates mirror lower levels of T_γ_ cells at the site of infection. Regulatory T cells (T_regs_) are represented as a fraction of T_γ_ in the model (for the details of the ODE system describing lymph node and blood dynamics, see Supplementary File 1), thus both outcomes are affected by the same mechanism (e.g., k_4_—CD4 precursor proliferation). Tables 3 and 5 emphasize a key protective role for both cytotoxic T-cell and T_γ_-cell responses in the lung (Figure 7a,b). However, these results suggest a more comprehensive role for CD8+ T cell priming and proliferation in regulating not only adaptive immune response magnitude in the blood and at the site of infection, but also on DC stimulation/maturation and trafficking. Mechanisms impacting blood outcomes are shown in Table 5 and Figure 7c,d. Here we see how most of the mechanisms elicit their effect early during infection (first 2 months post infection, as shown by the dotted vertical line in Figures 5–7), suggesting that the events happening right after the onset of the infection can shape a more protective response in the long term (which is ideal in a chronic infection such as tuberculosis).

Delaying trafficking of DCs to lymphatics and ultimately to the LN has a negative impact on all the memory T cell phenotypes in the blood (see *lungExitInterval* and *lymphExitInterval* mechanisms in Table 5 and Figure 7c,d). Again, this impact is important early during infection. Higher levels of resident DCs in the lung before infection are also important in establishing a more protective role for effector and effector memory T cell phenotypes in the blood (see Figure 7c,d). This latter result stresses again how early events are critical to establishing an effective and timely response during Mtb infection.

## 4. Discussion

A key step to mounting a protective immune response to Mtb and to most bacterial infection is represented by CD4+ and CD8+ T cell priming in lymph nodes. Facilitating migration of dendritic cells from the site of infection to the lymphatics, as well as enhancing trafficking of CD4+ and CD8+ effector T cells from the blood to the site of infection represent an important mechanism that could impact TB granuloma formation and function, and ultimately determine the outcome of TB infection in the host.

This study takes a multi-compartmental approach to studying antigen presentation, T cell priming, differentiation and trafficking in the context of TB granuloma formation. To better address these mechanisms, we built a new cell type, namely dendritic cell, into our existing multi-compartmental agent-based model of TB granuloma formation in the lung coupled to blood and lymph node dynamics [36]. This new model formulation allows us to better represent and investigate the impact of dendritic cell dynamics [20,22] on important aspects of immunity: antigen presentation, T cell priming, memory T cell generation, and ultimately into TB infection progression.

We successfully calibrated the model with non-human primate (NHP) experimental data on granulomas and bacterial levels in the lung, as well as longitudinal measures of memory T cell levels in the blood. The model is also able to recapitulate typical spatial distribution of cells within NHP granulomas in the lung (see Figure 3 in [36]).

The main conclusion of this study is that early events after initial Mtb infection are critical to establishing a timely and effective response. Although IFN-γ, macrophage activation and CD4+ T cells are necessary for mounting a protective response to Mtb [66], our results highlight an equally relevant role for CD8+ T cells, as suggested in previous experimental and modeling studies [67–69]. We show how we can lower bacterial burden and inflammation at the site of infection by enhancing either CD4+ or CD8+ T cell proliferation in the lymph node early on during infection (i.e., within the first 2 months). In some cases CD4+ and CD8+ T cells complement each other to achieve protection. For example, high CD8+ T cell proliferation rates in the lymph node result in overall lower levels of effector CD4+ T cells at the granuloma site (i.e., Tγ cells at the site of infection). In other words a larger cytotoxic T cell response (achieved by higher CD8+ T cell proliferation rates) can compensate for lower levels of Tγ cells at the site of infection.

Overall, T cell proliferation in the LN and T cell trafficking to the lung determine both the timing and magnitude of adaptive response at the site of infection and in the blood. Thus, identifying drugs that would enhance these processes could assist in the treatment of infection, as has been suggested in tumors [70].

By introducing dendritic cells into the model, we are able to better control both timing and magnitude of the mechanisms driving the adaptive T cell responses. In fact, we can negatively impact memory T cell phenotypes (both CD4+ and CD8+) by simply delaying dendritic cell trafficking to the draining lymph node. However, this outcome can only be achieved in the early stages of infection. This conclusion reinforces the working hypothesis that the best protective response to Mtb infection has to be mounted very early; otherwise the best outcome that can be achieved is a controlled chronic infection.

In the current model formulation, we describe cellular dynamics in the lymph node and blood compartments with a sufficient level of detail by a temporal-only representation (i.e., ODE system). However, with the introduction of dendritic cells in the model as agents, we are now working on implementing different subsets of Mtb-specificity in an ABM formulation of LNs [40,71] that can be used to replicate current vaccine clinical trials [72], as well as to test innovative immunotherapy strategies already used in cancer [73,74] but within the context of TB infection. It is this pairing of mathematical and computer modeling with experimental studies that has the greatest potential to push scientific discovery to the next level.

## Supplementary Material

Supplementary File 1, Supplementary File 2 and Supplementary File 3

## Figures and Tables

**Table 1:** Initial conditions. These values are based on the experimental data collected and published in our previous work [36]. The values and references for the scaling parameters (i.e., α, λ and host_LN (lymph node)) are given in Appendix A.

Variable	Value	Units	Description
APC	0	Cell count	Antigen presenting cell proxy in the lymph node
N_Ln,4_	N_B,4_ × (α/host_Ln)	Cell count	Mtb-specific LN Naïve CD4+ T cell
P_Ln,4_	0	Cell count	Mtb-specific LN Precursor CD4+ T cell
EM_Ln,4_	0	Cell count	Mtb-specific LN Effector Memory CD4+ T cell
CM_Ln,4_	0	Cell count	Mtb-specific LN Central Memory CD4+ T cell
N_B,4_	[255, 610] × λ	Cell/mm^3^	Mtb-specific Blood Naïve CD4+ T cell
E_B,4_	0	Cell/mm^3^	Mtb-specific Blood Effector CD4+ T cell
CM_B,4_	0	Cell/mm^3^	Mtb-specific Blood Central Memory CD4+ T cell
EM_B,4_	0	Cell/mm^3^	Mtb-specific Blood Effector Memory CD4+ T cell
N_Ln,8_	N_B,8_ × (α/host_Ln)	Cell count	Mtb-specific LN Naïve CD8+ T cell
P_Ln,8_	0	Cell count	Mtb-specific LN Precursor CD8+ T cell
EM_Ln,8_	0	Cell count	Mtb-specific LN Effector Memory CD8+ T cell
CM_Ln,8_	0	Cell count	Mtb-specific LN Central Memory CD8+ T cell
N_B,8_	[255, 610] × λ	Cell/mm^3^	Mtb-specific Blood Naïve CD8+ T cell
E_B,8_	0	Cell/mm^3^	Blood Effector CD8+ T cell
CM_B,8_	0	Cell/mm^3^	Blood Central Memory CD8+ T cell
EM_B,8_	0	Cell/mm^3^	Blood Effector Memory CD8+ T cell
N_Ln,nc4_	N_B,nc4_ × (α/host_Ln)	Cell count	Non-Mtb-specific LN Naïve CD4+ T cell
CM_Ln,nc4_	CM_B,nc4_ × (α/host_Ln)	Cell count	Non-Mtb-specific LN Central Memory CD4+ T cell
N_B,nc4_	[255, 610] × (1− λ)	Cell/mm^3^	Non-Mtb-specific Blood Naïve CD4+ T cell
E_B,nc4_	[47, 254] × (1 − λ)	Cell/mm^3^	Non-Mtb-specific Blood Effector CD4+ T cell
CM_B,nc4_	[83, 300] × (1 − λ)	Cell/mm^3^	Non-Mtb-specific Blood Central Memory CD4+ T cell
EM_B,nc4_	[50, 255] × (1 − λ)	Cell/mm^3^	Non-Mtb-specific Blood Effector Memory CD4+ T cell
N_Ln,nc8_	B_N,nc8_ × (α/host_Ln)	Cell count	Non-Mtb-specific LN Naïve CD8+ T cell
CM_Ln,nc8_	CM_N,nc8_ × (α/host_Ln)	Cell count	Non-Mtb-specific LN Central Memory CD8+ T cell
N_B,nc8_	[100, 672] ×(1 − λ)	Cell/mm^3^	Non-Mtb-specific Blood Naïve CD8+ T cell
E_B,nc8_	[43, 317] × (1 − λ)	Cell/mm^3^	Non-Mtb-specific Blood Effector CD8+ T cell
CM_B,nc8_	[36, 262] × (1 − λ)	Cell/mm^3^	Non-Mtb-specific Blood Central Memory CD8+ T cell
EM_B,nc8_	[11, 156] × (1 − λ)	Cell/mm^3^	Non-Mtb-specific Blood Effector Memory CD8+ T cell

**Table 2:** Significant Partial Rank Correlation Coefficients (PRCCs) for inflammation outcomes. List of all the parameters/mechanisms (rows) that have a significant (i.e., *p* < 10^−3^) PRCC with respect to outputs of the model that are directly related to some markers of infection (columns). See Appendix B for a detailed description of the outcomes analyzed here. A + (or −) indicates a positive (or negative) correlation between the parameter and the infection level outcome. The magnitude/strength of the correlation is given by the number of + (or −). The table recapitulates, whenever possible, the dynamics over time. The outputs with * are selected as examples to illustrate PRCC time courses (see Figure 5). Ext Mtb means extracellular bacteria that is not inside DCs or macrophages, Tot Mtb means total bacteria intracellular and extracellular, Gran size means actual measure of the granuloma diameter. The parameters τ define thresholds for T cell recruitment at each vascular source (in terms of number of molecules). The parameter λ represents the frequency of Mtb-specific Naïve T cells in the blood/LN (see Appendix A for details on all the parameters listed below).

	INFECTION (LUNG)				
Parameters	Tot Mtb*	Ext Mtb	Total Infected Macs*	Total Infected DCs*	Gran Size*
growthRateIntMtb	+ + +	+ + +	+ + +	+^1^	
lungExitInterval				+ + +	
$\tau_{T_{\gamma }-\mathrm{CC}}$—chemokine threshold for Tγ recruitment			++		
τ_Treg−TNF_—tumor necrosis factor (TNF) threshold for Treg recruitment					
k_4_—CD4+ T precursorproliferation	− − −	− − −		− −	
k_13_—CD8+ T precursorproliferation	− − −	− − −	− − −	− − −	early + then − − −
scalingMDC—Scaling to host factor representing the number of granulomas developing in the whole lung at time of infection					
k_11_—Naïve CD8+ T priming	− −	− −	− −	− −	− −
% of Resident DCs				+ +	^−^
λ		− −			

1 This PRCC is below 0.3, so it is not shown in Figure 5c.

**Table 3:** Significant PRCCs for inflammation outcomes. List of all the parameters/mechanisms (rows) that have a significant (i.e., *p* < 10^−3^) respect to outputs of the model that are directly related to inflammation (columns). See Appendix B for a detailed description of the outcomes analyzed here. A + (or −) indicates a positive (or negative) correlation between the parameter and the outcome. The magnitude/strength of the correlation is given by the number of + (or −). The table recapitulates, whenever possible, the dynamics over time. The outputs with * are selected as examples to illustrate PRCC time courses (see Figure 6). See Appendix A for details on the parameters listed below.

	INFLAMMATION (LUNG)				
PARAMETERS	Total Activated Macrophages	Tot Pet Hot^*^	Caseation/Necrosis	TNF*	IL10^*^
growthRateIntMtb		+ early^1^		+ + early	
$\tau_{T_{\gamma }-\mathrm{CC}}$—chemokine threshold for T_γ_ recruitment		− − early		− early^1^	+ + +
τ_Tcyt−CC_—chemokine threshold for Tcyt recruitment		− − early		− early then +^1^	+ + +
τ_Treg−CC_—chemokine threshold for Treg recruitment	−1	+ and then^1^		+ + early	− − −
k_2_—Naïve CD4 priming	+ +			− early^1^	+^1^
k_4_—CD4+ T precursor proliferation	+ + +				+^1^
k_13_—CD8+ T precursor proliferation	− − −	− − −	+ + + early − late	− − −	− − −
k_14_—CD8+ T differentiation—effector		+^1^		+^1^	
k_11_—Naïve CD8 priming		−	+ + early − late	−1	−1

1 These PRCCs are below 0.3, so they are not shown in Figure 6.

**Table 4:** Significant PRCCs for lung adaptive immune response outcomes. List of all the parameters/mechanisms (rows) that have a significant (i.e., *p* < 10^−3^) PRCC with respect to outputs of the model that are directly related to the adaptive immune response elicited in the lung, the site of infection (columns). See Appendix B for a detailed description of the outcomes analyzed here. A + (or −) indicates a positive (or negative) correlation between the parameter and the outcome. The magnitude/strength of the correlation is given by the number of + (or −). The table recapitulates, whenever possible, the dynamics over time. The outputs with * are selected as examples to illustrate PRCC time courses (see Figure 7a,b). See Appendix A for details on the parameters listed below.

	ADAPTIVE IMMUNE RESPONSE (LUNG)						
PARAMETERS	Mtb-Specific Tgam (Pro-Inflammatory) T Cells^*^	Mtb-Specific Tcyt^*^ (Cytotoxic) T Cells	Recruited Mtb-Specific Treg	Recruited Mtb-Specific Tcyt	DC Stimulated	DC Exited Lung	DC Exited Lymph
λ—Frequency of Mtb-specific Naïve T cells in the blood/LN		+ then −1					
k_11_—CD8 priming		+ + then −					
k_4_—CD4 precursor proliferation	+ + +		+ +				
k_13_—CD8 precursor proliferation	− −	+ + +	− − −	+ +	− − −	− − −	− − −
k_2_—CD4 priming	+ + + early						
% of Resident DCs					+ +	+ +	+ +
$\tau_{T_{\gamma }-\mathrm{CC}}$—chemokine threshold for Tγ recruitment		+ +^1^		+ +			
τ_Treg−CC_—chemokine threshold for Treg recruitment		− −1					

1 These PRCCs are below 0.3, so they are not shown in Figure 7.

**Table 5:** Significant PRCCs for blood adaptive immune response outcomes. List of all the parameters/mechanisms (rows) that have a significant (i.e., *p* < 10^−3^) PRCC with respect to outputs of the model that are directly related to Mtb-specific Memory T cell phenotypes in the blood compartment (columns). A + (or −) indicates a positive (or negative) correlation between the parameter and the outcome. The magnitude/strength of the correlation is given by the number of + (or −). The table recapitulates, whenever possible, the dynamics over time. The outputs with * are selected as examples to illustrate PRCC time courses (see Figure 7c,d). See Appendix A for details for the parameters listed below.

	BLOOD OUTCOMES—Mtb-Specific T Cells							
PARAMETERS	Naïve CD4	Effector CD4^*^	Central Memory CD4	Effector Memory CD4	Naïve CD8	Effector CD8^*^	Central Memory CD8	Effector Memory CD8
lungExitInterval		− − − early	− − − early	− − − early		− − − early	− − − early	− − − early
lymph_ExitInterval		− − − early	− − − early	− − − early		− − − early	− − − early	− − − early
% of Resident DCs		+ + early		+ + early		+ + + early		+ + + early
Initial Conditions for Mtb—specific Naïve CD4+ T cells—BLOOD	+ + +							
Initial Conditions for Mtb-specific Naïve CD8+ T cells—BLOOD					+ + +		+ + early	
host_LN—Number of involved lymph nodes in the host			− −				+ + early	
λ—Frequency of Mtb-specific Naïve T cells in the blood/LN	+ + +	+ + + early	+ + early	+ + early	+ + +	+ + + early	+ + + early	+ + + early
k_1_—Naïve CD4 recruitment rate	− − −	+ + + early	+ + early	+ + + early				
k_10_—Naïve CD8 recruitment rate					− − −	+ + + early	+ + + early	+ + + early
k_2_—Naïve CD4 priming	− − −	+ + + early	+ + early	+ + + early		+ + + early	+ + + early	+ + + early
k_11_—Naïve CD8 priming					− − −	+ + + early	+ + + early	+ + + early
k_4_—CD4 precursor proliferation		+ + +	+ + +	+ + +				
k_13_—CD8 precursor proliferation						+ + +	+ + +	+ + +
k_5_—Precursor CD4 differentiation to Effector rate	+ + +/−/+	− − −	+ + +/−/+					
k_14_—CD8 differentiation to effector						+ + +/−/+	− − −	+ + + early
μ_5_—Mature DC half-life in the LN						− − early		

## Appendix A

List of parameters and ranges for the computational model. Listed are the baseline parameter values used in the lymph node (LN), blood, and ordinary differential equation (ODE) compartments along with definitions and references to the values we chose. Those parameters marked with a * indicate they were calculated based upon the initial conditions of the system and the corresponding LN efflux term. These parameters were not varied during Latin hypercube sampling (LHS) experiments as they were constrained by a corresponding LN efflux parameter and the assumption that our initial conditions meet system homeostasis.

**Table:**

Parameter	Value	Units	Description	Reference
α	5.6 × 10^5^	μL	Conversion factor from Blood to Ln (max. blood volume)	Estimated and [36]
host_Ln	[1, 50]	count	Number of involved lymph nodes in the host	Estimated
λ	[10^−5^, 10^−3^]	“”	Frequency of Mycobacterium tuberculosis (Mtb)-specific Naïve T cells in the blood/LN	[75–77]
scalingMDC	[5, 15]	Count	Scaling to host factor representing the number of granulomas developing in the whole lung at time of infection	[52]
Sn4 *	N_LN,4_ × (α/host_Ln)	Cell/μL * day	Thymic output of Naïve CD4+ T cells	Estimated from Uncertainty Analysis
Sn8 *	N_LN,8_ × (α/host_Ln)	Cell/μL * day	Thymic output of Naïve CD8+ T cells	Estimated from Uncertainty Analysis
hs_1_	25	Cell count	Naïve CD4+ T cell recruitment half saturation	Estimated from Uncertainty Analysis
hs_4_	10	Cell count	Precursor CD4+ T cell proliferation half saturation	Estimated from Uncertainty Analysis
hs_5_	10	Cell count	Precursor CD4+ T cell differentiation half saturation	Estimated from Uncertainty Analysis
hs_8_	40	Cell count	Central Memory CD4+ T cell recruitment half saturation	Estimated from Uncertainty Analysis
hs_10_	25	Cell count	Naïve CD8+ T cell recruitment half saturation	Estimated from Uncertainty Analysis
hs_11_	10	Cell count	Naïve CD8+ T cell priming half saturation	Estimated from Uncertainty Analysis
hs_13_	10	Cell count	Precursor CD8+ T cell proliferation half saturation	Estimated from Uncertainty Analysis
hs_14_	10	Cell count	Precursor CD8+ T cell differentiation half saturation	Estimated from Uncertainty Analysis
hs_17_	157	Cell count	Central Memory CD8+ T cell recruitment half saturation	Estimated from Uncertainty Analysis
k_1_	[5 × 10^−3^, 1]	day^−1^	Naïve CD4+ T cell recruitment rate	Estimated from Uncertainty Analysis
k_2_	[10^−6^, 10^−1^]	day^−1^	Naïve CD4+ T cell Priming rate	Estimated from Uncertainty Analysis
k_3_	[10^−7^, 10^−2^]	day^−1^	Central Memory CD4+ T cell reactivation rate	Estimated from Uncertainty Analysis
k_4_	[10^−2^, 1.2]	day^−1^	Precursor CD4+ T cell proliferation rate	Estimated from Uncertainty Analysis
k_5_	[0.01, 0.75]	day^−1^	Precursor CD4+ T cell differentiation to Effector rate	Estimated from Uncertainty Analysis
k_6_	0.001	day^−1^	Precursor CD4+ T cell differentiation to Central Memory	Estimated from Uncertainty Analysis
k_7_	[0.05, 0.75]	day^−1^	Effector CD4+ T cell differentiation to Effector Memory	Estimated from Uncertainty Analysis
k_8_	[0.1, 0.5]	day^−1^	Central Memory CD4+ T cell recruitment rate	Estimated from Uncertainty Analysis
k_10_	[5 × 10^−3^, 1]	day^−1^	Naïve CD8+ T recruitment cell rate	Estimated from Uncertainty Analysis
k_11_	[10^−6^, 10^−1^]	day^−1^	Naïve CD8+ T cell priming rate	Estimated from Uncertainty Analysis
k_12_	[10^−7^, 10^−2^]	day^−1^	Central Memory CD8+ T cell reactivation rate	Estimated from Uncertainty Analysis
k_13_	[10^−2^, 1.2]	day^−1^	Precursor CD8+ T cell proliferation rate	Estimated from Uncertainty Analysis
k_14_	[0.01, 0.75]	day^−1^	Precursor CD8+ T cell differentiation to Effector rate	Estimated from Uncertainty Analysis
k_15_	0.001	day^−1^	Precursor CD8+ T cell differentiation to Central Memory	Estimated from Uncertainty Analysis
k_16_	[0.05, 0.75]	day^−1^	Effector CD8+ T cell differentiation to Effector Memory	Estimated from Uncertainty Analysis
k_17_	[0.05, 0.75]	day^−1^	Central Memory CD8+ T cell recruitment rate	Estimated from Uncertainty Analysis
μ_1_	0.2	day^−1^	Effector CD4+ T cell death rate	[19,20,22,23,36]
μ_2_	0.04	day^−1^	Effector Memory CD4+ T cell death rate	[19,20,22,23,36]
μ_3_	0.2	day^−1^	Effector CD8+ T cell death rate	[19,20,22,23,36]
μ_4_	0.04	day^−1^	Effector Memory CD8+ T cell death rate	[19,20,22,23,36]
μ_5_	[0.1, 1]	day^−1^	APC death rate	[19,20,22,23,36]
μ_6_	0.1	day^−1^	Precursor CD4+ T cell death rate	[19,20,22,23,36]
μ_7_	0.1	day^−1^	Precursor CD8+ T cell death rate	[19,20,22,23,36]
μ_8_ *	3.93 × 10^−4^	day^−1^	Naïve CD4+ T cell death rate	
μ_9_ *	2.27 × 10^−4^	day^−1^	Naïve CD8+ T cell death rate	
ρ_1_	3 × 10^8^	Cell count	Precursor carrying capacity	[19,20,22,23,36]
Wp^4^	0.735	“”	Weight factor for Precursor CD4+ T in CD8+ T cell priming	Estimated from Uncertainty Analysis
ξ_1_ *	ξ_2_ × (N_Ln,nc4_/N_B,nc4)_/α	day^−1^	Naïve CD4 Lymph Influx	
ξ_2_	[0.6, 1]	day^−1^	Naïve CD4 Lymph Efflux	Estimated from Uncertainty Analysis
ξ_3_	[2, 5]	day^−1^	Effector CD4 Lymph Efflux	Estimated from Uncertainty Analysis
ξ_4_ *	ξ_5_ × (CM_Ln,nc4_/CM_B,nc4_)/α	day^−1^	Central Memory CD4 Lymph Influx	
ξ_5_	0.489	day^−1^	Central Memory CD4 Lymph Efflux	Estimated from Uncertainty Analysis
ξ_6_	[2, 5]	day^−1^	Effector Memory CD4 Lymph Efflux	Estimated from Uncertainty Analysis
ξ_7_ *	ξ_8_ × (N_Ln,nc8_/N_B,nc8_)/α	day^−1^	Naïve CD8 Lymph Influx	
ξ_8_	[0.6, 1]	day^−1^	Naïve CD8 Lymph Efflux	Estimated from Uncertainty Analysis
ξ_9_	[2, 5]	day^−1^	Effector CD8 Lymph Efflux	Estimated from Uncertainty Analysis
ξ_10_ *	ξ_11_ × (CM_Ln,nc8_/CM_B,nc8_)/α	day^−1^	Effector CD8 Lymph Influx	
ξ_11_	[2, 5]	day^−1^	Central Memory CD8 Lymph Efflux	Estimated from Uncertainty Analysis
ξ_12_	[2, 5]	day^−1^	Effector Memory CD8 Lymph Efflux	Estimated from Uncertainty Analysis
*proliferationTime*	8	h	Doubling time for cognate T cells in the lung	[40,71]
*maxDivisions*	4	− −	Max number of divisions for T cells in the lung	[40,71]
τ_Tγ_ −CC	[1, 20]	# molecules	Chemokine threshold for T_γ_ recruitment	Estimated from Uncertainty Analysis
τ_Tγ_−TNF	[1, 5]	# molecules	Tumor necrosis factor (TNF) threshold for T_γ_ recruitment	Estimated from Uncertainty Analysis
τ_TCyt_*^–^*CC	[1, 20]	# molecules	Chemokine threshold for Tcyt recruitment	Estimated from Uncertainty Analysis
τ_TCyt−TNF_	[1, 5]	# molecules	TNF threshold for Tcyt recruitment	Estimated from Uncertainty Analysis
τ_Treg_^−^CC	[1, 10]	# molecules	Chemokine threshold for Treg recruitment	Estimated from Uncertainty Analysis
τ_Treg−TNF_	[1, 5]	# molecules	TNF threshold for Treg recruitment	Estimated from Uncertainty Analysis
*ProbKillMac*	[0.05, 0.21]	probability	Probability of Tcyt to kill Macs	[19,20,22,23,36]
*probKillMacCleanly*	[0.15, 0.31]	probability	Probability of Tcyt to kill Macs and all their intracellular Mtb load	[19,20,22,23,36]
*probApoptosisFasFasL*	[0.001, 0.02]	probability	Probability of undergoing apotposis induced by Tγ	[19,20,22,23,36]
*lungExitInterval*	[6, 144]	10 min	Time it takes a stimulated dendritic cell (DC) to exit the lung through lymphatics	[19,20,22]
*lymphaticsExitInterval*	[6, 40]	10 min	Time a DC takes to traffic through the lymphatics and reach the lymph node (LN)	[19,20,22]
*percentOfMacInitNumber*	[0.05, 0.25]	%, and used as probability as well	Percentages of DCs that populates the grid initially (calculated as a percentage of initial resident macrophages). It is also used for recruitment on new DC into the grid, at the time a macrophage is recruited	[20,22]
*growthRateIntMtb*	[1.0029, 1.0035]	10 min	Doubling time of intracellular Mtb	[23]
*growthRateExtMtb*	[1.00124, 1.0014]	10 min	Doubling time of extracellular Mtb	[23]

## Appendix B

List of all the outcomes of interest analyzed during our uncertainty and sensitivity analysis.

**Table:**

Outcome of Interest	Compartment	Definition
**Inflammation**		

‘TotalMr’	Lung	Total Resting Macrophages
‘TotalDCellMr’	Lung	Total Unstimulated Dendritic Cells
‘TotalMa’	Lung	Total Activated Macrophages
‘TotPethot’	Lung	Total Pet Hot reading from the PET-CT scan
‘NrCaseated’	Lung	Number of caseated compartments in the granuloma
‘TNF’	Lung	Tumor Necrosis Factor molecues
‘IL10’	Lung	Interlukin 10 molecules

**Infection**		

‘TotMtb’	Lung	Total Mycobacterium tuberculosis (Mtb) burden
‘IntMtb’	Lung	Intracellular Mtb burden
‘ExtMtb’	Lung	Extracellular Mtb burden
‘repExtMtb’	Lung	Extracellular replicating Mtb burden
‘NonReplExtMtb’	Lung	Extracellular non-replicating Mtb burden
‘TotalMi’	Lung	Total Infected Macrophages
‘TotalMci’	Lung	Total Chronically Infected Macrophages
‘TotalDCellMi’	Lung	Total Infected Dendritic Cells
‘TotalDCellMci’	Lung	Total Chronically Infected Dendritic Cells
‘LesionSize’	Lung	Diameter of the granuloma lesion

**Table:**

Adaptive Immune Response	Compartment	Definition
‘TγCognate’	Lung	Number of Mtb-specific Tγ cells present in the lung
‘TcytCognate’	Lung	Number of Mtb-specific Tcyt cells present in the lung
‘TgamRecruitedCognate’	Lung	Number of Mtb-specific Tγ cells recruited to the lung
‘TcytRecruitedCognate’	Lung	Number of Mtb-specific Tcyt cells recruited to the lung
‘DCellStimulated’	Lung	Number of Dendritic Cells that have been stimulated
‘DCellExitedLung’	Lung→lymphatics	Number of Dendritic Cells that have left the lung upon stimulation
‘DCellExitedLymphatics’	Lymphatics→LN	Number of Dendritic Cells that have left the lymphatics to enter the lymph node
‘BlN4C’	Blood	Concentration of Mtb-Specific Naïve CD4+ T cells
‘BlE4C’	Blood	Concentration of Mtb-Specific Effector CD4+ T cells
‘BlCM4C’	Blood	Concentration of Mtb-Specific Central Memory CD4+ T cells
‘BlEM4C’	Blood	Concentration of Mtb-Specific Effector Memory CD4+ T cells
‘BlN8C’	Blood	Concentration of Mtb-Specific Naïve CD8+ T cells
‘BlE8C’	Blood	Concentration of Mtb-Specific Effector CD8+ T cells
‘BlCM8C’	Blood	Concentration of Mtb-Specific Central Memory CD8+ T cells
‘BlEM8C’	Blood	Concentration of Mtb-Specific Effector Memory CD8+ T cells
‘APC’	Lymph node	Number of Dendritic Cells in the Lymph Node [LN]
‘LnN4C’	Lymph node	Number of Mtb-Specific Naïve CD4+ T cells
‘LnP4C’	Lymph node	Number of Mtb-Specific Precursor CD4+ T cells
‘LnE4C’	Lymph node	Number of Mtb-Specific Effector CD4+ T cells
‘LnCM4C’	Lymph node	Number of Mtb-Specific Central Memory CD4+ T cells
‘LnEM4C’	Lymph node	Number of Mtb-Specific Effector Memory CD4+ T cells
‘LnN8C’	Lymph node	Number of Mtb-Specific Naïve CD8+ T cells
‘LnP8C’	Lymph node	Number of Mtb-Specific Precursor CD8+ T cells
‘LnE8C’	Lymph node	Number of Mtb-Specific Effector CD8+ T cells
‘LnCM8C’	Lymph node	Number of Mtb-Specific Central Memory CD8+ T cells
‘LnEM8C’	Lymph node	Number of Mtb-Specific Effector Memory CD8+ T cells

## Abbreviations

The following abbreviations are used in this manuscript:

TB: tuberculosis
Mtb: Mycobacterium tuberculosis
LN: lymph node
