# Supplementary material for: A Multi-Compartment Hybrid Computational Model Predicts Key Roles for Dendritic Cells in Tuberculosis Infection
Source: Computation (Basel). Author manuscript; Available in PMC 2017 Oct 4. (PMC5627612; doi:10.3390/computation4040039)
Supplement: Supplementary File 1, Supplementary File 2 and Supplementary File 3 [file NIHMS856047-supplement-Supplementary_File_1__Supplementary_File_2_and_Supplementary_File_3.zip › Supplementary File 1.pdf]

Supplementary Materials: A Multi-Compartment Hybrid Computational Model Predicts Key Roles for Dendritic Cells in Tuberculosis Infection

Simeone Marino and Denise E. Kirschner

Supplementary File 1 (adjusted from our previous manuscript [1]).

Mathematical Model Equations of the Blood and Lymph Node Compartments

The next section describes in detail how we developed an ordinary differential equation model for CD4+ *Mycobacterium tuberculosis* (Mtb)-specific T cell dynamics.

Measure units are the cell count in the lymph compartment and the cells/mm[3] in the blood compartment. The term α represents the volume of blood in μL and is used for scaling cells when they traffic between the blood compartment and the lymph compartment.

Antigen presentation and priming in the lymph node compartment is driven by the following equation

$$\frac{dDC}{dt} = -\mu_5 DC \tag{S1}$$

which tracks Dendritic Cells (DCs) based on the number of DCs that exit the lymphatics and enter the lymph node (see Sections 2.3 and 2.4 of the main manuscript for details).

Naïve T cells (Equation (S2)) are recruited to the lymph node at a rate (k_1) dependent on cytokine production in the lymph node. Since we do not track cytokines in the lymph node model, we use DCs also as a proxy for cytokine production (modeled as a Michaelis–Menten term in Equation (S2)). Other terms included basal influx (ξ_1) and efflux (ξ_2), as well as mass action priming to precursor cells (k_2).

$$\frac{dN_4^{LN}}{dt} = \alpha\left(k_1 N_4^B\left(\frac{DC}{DC+hs_1}\right)+\xi_1 N_4^B\right)-\xi_2 N_4^{LN}-k_2 N_4^{LN} DC \tag{S2}$$

Precursor CD4+ T cells (Equation (S3)) are generated through priming of Mtb-specific naïve T cells as well as through re-activation of Mtb-specific central memory T cells; both processes are expressed as mass action terms. Proliferation is modeled as logistic growth.

$$\begin{aligned}\frac{dP_4^{LN}}{dt} &= DC\left(k_2 N_4^{LN}+k_3 CM_4^{LN}\right)+k_4 P_4^{LN}\left(1-\left(\frac{P_4^{LN}}{\rho_1}\right)\right)\left(\frac{DC}{DC+hs_4}\right)\\ &-k_5 P_4^{LN}\left(\frac{DC}{DC+hs_5}\right)-k_6 P_4^{LN}\left(1-\left(\frac{DC}{DC+hs_5}\right)\right)-\mu_6 P_4^{LN}\end{aligned} \tag{S3}$$

A Michaelis–Menten term based on antigen stimulation (DC levels) was used to adjust proliferation (k_4) and differentiation rates (k_5 and k_6). The likelihood of precursor cells differentiating into effector cells is directly proportional to the amount of antigen stimulation (k_5). The opposite assumption was applied to the likelihood of precursor cells differentiating into central memory (k_6). A death term (μ_6) ensured that the precursor population did not persist in the absence of infection. No precursor population exits the lymph node. Effector CD4+ T cells are modeled in Equation (S4).

$$\frac{dE_4^{LN}}{dt} = k_5 P_4^{LN}\left(\frac{DC}{DC + hs_5}\right) - \xi_3 E_4^{LN} - k_7 E_4^{LN} \quad \text{(S4)}$$

Terms in the equation include efflux to the blood (ξ_3), and a linear differentiation to the effector memory T cell population (k_7). We assumed that no effector T cells die in the lymph node (they can die in the blood before entering the lung) and that no migration occurs directly from the blood. Similarly to naïve cells, central memory T cells (Equation (S5)) are recruited to the lymph node (k_8) in addition to a basal influx rate (ξ_4). Other terms include differentiation from precursor cells (k_6), reactivation to precursor cells (k_3) and efflux into the blood (ξ_4). Given their relatively long lifespan compared to the length of the in silico simulation (i.e., 200 days) we do not have a death term in Equation (S5).

$$\frac{dCM_4^{LN}}{dt} = \alpha\left(k_8 CM_4^B\left(\frac{DC}{DC + hs_8}\right) + \xi_4 CM_4^B\right) + k_6 P_4^{LN}\left(1 - \left(\frac{DC}{DC + hs_5}\right)\right) - \\ - k_3 CM_4^{LN} DC - \xi_5 CM_4^{LN} \quad \text{(S5)}$$

Effector memory cell formation is described in Equation (S6). A linear term captures the differentiation of CD4+ effector T cells into CD4+ effector memory (k_7). The last term represented efflux to the blood (ξ_6). Given their long life span, we do not account for cell death in Equation (S6). Like effector T cells, effector memory T cells do not enter the lymph node directly from the blood.

$$\frac{dEM_4^{LN}}{dt} = k_7 E_4^{LN} - \xi_6 EM_4^{LN} \quad \text{(S6)}$$

For the blood compartment we track four different T cell Mtb-specific phenotypes. The Mtb-specific naïve CD4+ T cell blood population is modeled by Equation (S7). We have terms for a constant source supplied from the thymus (multiplied by the Mtb-specific frequency λ, i.e., λs_{N_4}) to track specific and non-specific cells, migration from the lymph node (ξ_2), extra recruitment to the lymph node (k_1), migration to the lymph node (ξ_1), and death (μ_8).

$$\frac{dN_4^B}{dt} = \lambda s_{N_4} + \alpha^{-1}\xi_2 N_4^{LN} - k_1 N_4^B\left(\frac{DC}{DC + hs_1}\right) - \xi_1 N_4^B - \mu_8 N_4^B \quad \text{(S7)}$$

The values for s_{N_4} and μ_8 (as well as s_{N_8} and μ_9 later for CD8+ T cells) are chosen to maintain equilibrium in the total Naïve T cell populations (based on the initial conditions taken from the Non-Human Primate; NHP, blood data). Equation (S8) describes effector CD4+ T cells dynamics in the blood with two terms: migration from the lymph node (ξ_3) and death. Effector T cells are recruited to the lung during each agent-based model (ABM) time step. This loss was implicated as a change in the initial conditions before each ODE time step.

$$\frac{dE_4^B}{dt} = \alpha^{-1}\xi_3 E_4^{LN} - \mu_1 E_4^B \quad \text{(S8)}$$

Central memory cells in the blood (Equation (S9)) migrate from (ξ_5) and to the lymph node (ξ_4). Central memory cells are not recruited to the site of infection.

$$\frac{dCM_4^B}{dt} = \alpha^{-1}\xi_5 CM_4^{LN} - \xi_4 CM_4^B - k_8 CM_4^B\left(\frac{DC}{DC+hs_8}\right) \quad \text{(S9)}$$

Effector memory cells in the blood, as shown in Equation (S10), are modeled by two terms: migration from the lymph node (ξ_6) and death. Similar to effector cells these were recruited to the site of infection.

$$\frac{dEM_4^B}{dt} = \alpha^{-1}\xi_6 EM_4^{LN} - \mu_2 EM_4^B \quad \text{(S10)}$$

Lymph Node and Blood Compartment Equations for Mtb-Specific CD8+ T Cells

We modeled dynamics of Mtb-specific CD8+ T cell processes in the lymph node and blood in a manner identical to how we did for CD4+ T cells, with the exception of few terms and different parameter values. One such exception is the priming of Mtb-specific naïve CD8+ T cells in the lymph node (LN), which is affected by cytokines released by activated CD4+ T cells in the lymph node. This is modeled by a Michaelis–Menten term including activated CD4+ T effector cells and a weighted term for CD4+ precursor T cells (as shown in the Equations (S11) and (S12), respectively Mtb-specific naïve and precursor CD8+ T cells in the LN).

$$\begin{aligned}\frac{dN_8^{LN}}{dt} &= \alpha\left(k_{10}N_8^B\left(\frac{DC}{DC+hs_{10}}\right) + \xi_7 N_8^B\right) - \xi_8 N_8^{LN} - \\ &-k_{11}N_8^{LN}DC\left(\frac{[E_4^{LN}+W_{P_4}P_4^{LN}]}{[E_4^{LN}+W_{P_4}P_4^{LN}]+hs_{11}}\right)\end{aligned} \quad \text{(S11)}$$

$$\begin{aligned}\frac{dP_8^{LN}}{dt} &= k_{11}N_8^{LN}DC\left(\frac{[E_4^{LN}+W_{P_4}P_4^{LN}]}{[E_4^{LN}+W_{P_4}P_4^{LN}]+hs_{11}}\right) + k_{12}CM_8^{LN}DC \\ &+k_{13}P_8^{LN}\left(1-\frac{P_4^{LN}+P_8^{LN}}{\rho_1}\right)\left(\frac{DC}{DC+hs_{13}}\right) - k_{14}P_8^{LN}\left(\frac{DC}{DC+hs_{14}}\right) - \\ &-k_{15}P_8^{LN}\left(1-\left(\frac{DC}{DC+hs_{14}}\right)\right) - \mu_7 P_8^{LN}\end{aligned} \quad \text{(S12)}$$

The equations for Mtb-specific CD8+ T cells in the lymph node and blood compartments are described below.

Effector CD8+ - lymph node (LN)

$$\frac{dE_8^{LN}}{dt} = k_{14}P_8^{LN}\left(\frac{DC}{DC+hs_{14}}\right) - \xi_9 E_8^{LN} - k_{16}E_8^{LN} \quad \text{(S13)}$$

Central Memory CD8+ - LN

$$\frac{dCM_8^{LN}}{dt} = \alpha\left(k_{17}CM_8^B\left(\frac{DC}{DC+hs_{17}}\right)+\xi_{10}CM_8^B\right)+ k_{15}P_8^{LN}\left(1-\left(\frac{DC}{DC+hs_{14}}\right)\right)-k_{12}CM_8^{LN}DC-\xi_{11}CM_8^{LN} \tag{S14}$$

Effector Memory CD8+ - LN

$$\frac{dEM_8^{LN}}{dt} = k_{16}E^{LN}_8 - \xi_{12}EM_8^{LN} \tag{S15}$$

Naïve CD8+ - Blood

$$\frac{dN_8^B}{dt} = \lambda s_{N_8} + \alpha^{-1}\xi_8 N_8^{LN} - k_{10}N_8^B\left(\frac{DC}{DC+hs_{10}}\right) - \xi_7 N_8^B - \mu_9 N_8^B \tag{S16}$$

Effector CD8+ - Blood

$$\frac{dE_8^B}{dt} = \alpha^{-1}\xi_9 E_8^{LN} - \mu_3 E_8^B \tag{S17}$$

As with CD4+ effector cells in the blood, recruitment to the site of infection was modeled by changing the initial conditions before each ordinary differential equation (ODE) time step.

Central Memory CD8+ - Blood

$$\frac{dCM_8^B}{dt} = \alpha^{-1}\xi_{11}CM_8^{LN} - \xi_{10}CM_8^B - k_{17}CM_8^B\left(\frac{DC}{DC+hs_{17}}\right) \tag{S18}$$

Effector Memory CD8+ - Blood

$$\frac{dEM_8^B}{dt} = \alpha^{-1}\xi_{12}EM_8^{LN} - \mu_4 EM_8^B \tag{S19}$$

Non Mtb-Specific CD4+ and CD8+ Lymphocytes

Our computational model keeps track of non Mtb-specific T cells similarly to their respective Mtb-specific (antigen specific) counterparts. However, non Mtb-specific T cells do not respond to antigen, therefore, no priming occurs in any cell population and no precursor cells are generated. Also, since we assume neither effector nor effector memory T cells enter the lymph compartment from the blood, we do not model effector or effector memory cell populations within the lymph node compartment (as shown in Figure 2B). The production of the non-tuberculosis (TB)-specific effector cells was modeled as a source term in the blood compartment and was included to meet the assumption that the pre-infection data describes

homeostasis. The equations for non-Mtb-specific CD4+ and CD8+ T cells are shown below. Moreover, including non-Mtb-specific cells at the site of infection makes model predictions more realistic due to the total cell numbers more accurately reflecting the actual numbers in blood.

Naïve CD4+ non Mtb-Specific – LN

$$\frac{dN_{nc4}^{LN}}{dt} = \alpha\left(k_1 N_{nc4}^{B}\left(\frac{DC}{DC + hs_1}\right) + \xi_1 N_{nc4}^{B}\right) - \xi_2 N_{nc4}^{LN} \quad \text{(S20)}$$

Central Memory CD4+ non Mtb-Specific – LN

$$\frac{dCM_{nc4}^{LN}}{dt} = \alpha\left(k_8 CM_{nc4}^{B}\left(\frac{DC}{DC + hs_8}\right) + \xi_4 CM_{nc4}^{B}\right) - \xi_5 CM_{nc4}^{LN} \quad \text{(S21)}$$

Naïve CD4+ non Mtb-Specific – Blood

$$\frac{dN_{nc4}^{B}}{dt} = (1-\lambda)s_{N_4} + \alpha^{-1}\xi_2 N_{nc4}^{LN} - k_1 N_{nc4}^{B}\left(\frac{DC}{DC + hs_1}\right) - \xi_1 N_{nc4}^{B} - \mu_8 N_{nc4}^{B} \quad \text{(S22)}$$

Effector CD4+ non Mtb-Specific – Blood

$$\frac{dE_{nc4}^{B}}{dt} = s_{E_{nc4}} - \mu_1 E_{nc4}^{B} \quad \text{(S23)}$$

As non Mtb-specific effector cells in the blood must be produced somewhere in the body, they are modeled as source and a death rate equal to that of their Mtb-specific counterparts

Central Memory CD4+ non Mtb-Specific – Blood

$$\frac{dCM_{nc4}^{B}}{dt} = \alpha^{-1}\xi_5 CM_{nc4}^{LN} - \xi_4 CM_{nc4}^{B} - k_8 CM_{nc4}^{B}\left(\frac{DC}{DC + hs_8}\right) \quad \text{(S24)}$$

Effector Memory CD4+ non Mtb-Specific – Blood

$$\frac{dEM_{nc4}^{B}}{dt} = s_{EM_{nc4}} - \mu_2 EM_{nc4}^{B} \quad \text{(S25)}$$

As was the case with non Mtb-specific cells, CD8+ T cell processes are modeled identical to their CD4+ counterparts.

Naïve CD8+ non Mtb-Specific – LN

$$\frac{dN_{nc8}^{LN}}{dt} = \alpha\left(k_{10}N_{nc8}^{B}\left(\frac{DC}{DC+hs_{10}}\right)+\xi_7 N_{nc8}^{B}\right)-\xi_8 N_{nc8}^{LN} \tag{S26}$$

Central Memory CD8+ non Mtb-Specific – LN

$$\frac{dCM_{nc8}^{LN}}{dt} = \alpha\left(k_{17}CM_{nc8}^{B}\left(\frac{DC}{DC+hs_{17}}\right)+\xi_{10} CM_{nc8}^{B}\right)-\xi_{11} CM_{nc8}^{LN} \tag{S27}$$

Naïve CD8+ non Mtb-Specific – Blood

$$\frac{dN_{nc8}^{B}}{dt} = (1-\lambda)s_{N_4} + \alpha^{-1}\xi_8 N_{nc8}^{LN} - k_{10}N_{nc8}^{B}\left(\frac{DC}{DC+hs_{10}}\right)-\xi_7 N_{nc8}^{B} - \mu_9 N_{nc8}^{B} \tag{S28}$$

Effector CD8+ non Mtb-Specific – Blood

$$\frac{dE_{nc8}^{B}}{dt} = s_{E_{nc8}} - \mu_3 E_{nc8}^{B} \tag{S29}$$

Central Memory CD8+ non Mtb-Specific – Blood

$$\frac{dCM_{nc8}^{B}}{dt} = \alpha^{-1}\xi_{11} CM_{nc8}^{LN} - \xi_{10} CM_{nc8}^{B} - k_{17}CM_{nc8}^{B}\left(\frac{DC}{DC+hs_{17}}\right) \tag{S30}$$

Effector Memory CD8+ non Mtb-Specific – Blood

$$\frac{dEM_{nc8}^{B}}{dt} = s_{EM_{nc8}} - \mu_4 EM_{nc8}^{B} \tag{S31}$$

References

1. Marino, S.; Gideon, H.P.; Gong, C.; Mankad, S.; McCrone, J.T.; Lin, P.L.; Linderman, J.J.; Flynn, J.L.; Kirschner, D.E. Computational and Empirical Studies Predict Mycobacterium Tuberculosis-Specific T Cells as a Biomarker for Infection Outcome. *PLoS Comput. Biol.* **2016**, *12*, doi:10.1371/journal.pcbi.1004804.
